# Fluoroscopy-free Resuscitative Endovascular Balloon Occlusion of the Aorta (REBOA) for controlling life threatening postpartum hemorrhage

**DOI:** 10.1371/journal.pone.0174520

**Published:** 2017-03-29

**Authors:** Knut Haakon Stensaeth, Edmund Sovik, Ingrid Natasha Ylva Haig, Erna Skomedal, Arve Jorgensen

**Affiliations:** 1 Dept of Radiology and Nuclear Medicine, St Olavs University Hospital, Trondheim, Norway; 2 Institute of Circulation and Medical Imaging, Norwegian University of Science and Technology, Trondheim, Norway; 3 Dept of Radiology and Nuclear Medicine, Oslo University Hospital Ullevaal, Oslo, Norway; 4 Dept of Radiology and Nuclear Medicine, Stavanger University Hospital, Stavanger, Norway; Azienda Ospedaliero Universitaria Careggi, ITALY

## Abstract

**Background:**

Severe postpartum hemorrhage occurs in 1/1000 women giving birth. This condition is often dramatic and may be life threatening. Resuscitative Endovascular Balloon Occlusion of the Aorta (REBOA) has in recent years been introduced as a novel treatment for hemorrhagic shock. We present a series of fluoroscopy-free REBOA for controlling life threatening postpartum hemorrhage.

**Methods:**

In 2008 an ‘aortic occlusion kit’ was assembled and used in three Norwegian university hospitals. The on-call interventional radiologist (IR) was to be contacted with a response time < 30 minutes in case of life threatening PPH. Demographics and characteristics were noted from the medical records.

**Results:**

This retrospective study includes 36 patients treated with fluoroscopy-free REBOA for controlling severe postpartum hemorrhage in the years 2008–2015. The REBOA success rate was 100% and no patients died from REBOA related complications. Uterine artery embolization was performed in 17 (47%) patients and a hysterectomy in 16 (44%) patients. A short (11cm) introducer length was strongly associated with iliac artery thrombus formation (*ρ* = 0.50, *P* = 0.002). In addition, there was a strong negative correlation between uterine artery embolization and hysterectomy (*ρ* = -0.50, *P* = 0.002).

**Conclusions:**

Our Norwegian experience indicates the clinical safety and feasibility of REBOA in life threatening PPH. Also, REBOA can be used in an emergency situation without the use of fluoroscopy with a high degree of technical success. It is important that safety implementation of REBOA is established, especially through limited aortic balloon occlusion time and a thorough balloon deflation regime.

## Introduction

Hemorrhagic shock is very challenging and is a leading cause of death in trauma patients worldwide [1]. Appropriate management of the massively bleeding patient includes early identification of bleeding sources followed by prompt measures to minimize blood loss, restore tissue perfusion, and achieve hemodynamic stability. Where hemorrhage is controlled expeditiously, patients often recover with little to no morbidity [2].

Severe postpartum hemorrhage (PPH) occurs in 1/1000 women giving birth [3], and is defined by a total blood loos > 1000 ml in the first 24 hours after delivery. This condition is often dramatic and is the leading cause of pregnancy-related death in the USA [2]. Medical approaches to severe PPH include treatments with intravenous fluids and blood products, the use of uterotonic medications such as oxytocin, as well as external uterine massage, curettage and/or an intrauterine balloon. However, the evolution of endovascular techniques has established a role for arterial embolization in the setting of uncontrolled PPH. Embolization of the uterine arteries has the advantages of being a minimally invasive treatment that preserves the uterus without eliminating the possibility of future surgical intervention if necessary [3].

Resuscitative Endovascular Balloon Occlusion of the Aorta (REBOA), which was developed by Edwards et al. in 1953 [4], was initially intended for surgical treatment of abdominal aortic aneurysms and later applied to traumatic hemorrhagic shock [5]. There have been several reports on the use of REBOA in recent years, both for surgical bleeding [6], as well as a temporary measure in Caesarean hysterectomy for placenta percreta [7]. In an emergency situation fluoroscopy is commonly not available, and to achieve immediate control of severe PPH fluoroscopy-free REBOA is therefore often needed. Only a few single case reports on the use of REBOA in PPH have been reported so far [6–8], in addition to our first report on six patients [9].

Fluoroscopy-free REBOA was introduced in Norway in 2008, and has been performed in three major university hospitals since then. The intention was to introduce a lifesaving procedure with few complications that was easy to perform in an emergency situation. To our knowledge, this is the largest reported series of fluoroscopy-free REBOA to control life threatening PPH.

## Materials and methods

### Patients and study design

This retrospective study includes all patients, vaginal and caesarean birth (n = 36), treated with fluoroscopy-free REBOA for severe PPH in three major university hospitals in Norway in the years 2008–2015. The contributing hospitals were St. Olavs Trondheim University Hospital (n = 13), Oslo University Hospital, Ullevaal, (n = 13) and Stavanger University Hospital (n = 10). The study was approved by the Regional Ethics Committee, and passive consent was obtained from all included patients.

### Aortic balloon occlusion kit

In 2008 an ‘aortic occlusion kit’ was assembled that consisted of different sized PTS-X semi-compliant balloons with diameters ranging from 15 to 30 mm (NuMED Canada Inc., Cornwall, ON, Canada), 6–8 French introducers 11 and 23 cm long (Cordis Corporation, Hialeah, FL, USA), and J-curved fixed core 150 cm 0.035-inch steel guidewires (Boston Scientific, Marlborough, MA, and Argon Medical Devices, Plano, TX, USA). Table 1 shows the main equipment of the kit that was used during the 36 REBOA procedures.

**Table 1:** Main content of the “aortic occlusion kit” used in all hospitals.

	n = 36
Introducer size, French	8 (6–8)
Introducer length, cm	11 (11–23)
Guidewire size, inches	0.035
Guidewire length, cm	150
Balloon diameter, mm	25 (15–30)
Balloon length, mm	30 (30–40)
Balloon shaft length, cm	80

Data are presented as median (range). Individual adjustments to introducer and balloon sizes were done.

### Intervention

The indication for REBOA was hemodynamically unstable severe PPH, and the on-call interventional radiologist (IR) was to be contacted with a response time < 30 min. All hospitals had a similar REBOA procedure, which has been described previously [9], and all procedures were done by the interventional radiologist on call by using the Seldinger technique through a needle puncture in the common femoral artery under ultrasound guidance due to weak femoral pulses. The balloon catheter was inserted through the introducer over the guidewire approximately 30 cm into the abdominal artery and the balloon was then inflated with isotonic saline (using a 20 ml syringe) until a slight resistance was felt (approximately 7–8 ml for 20 mm balloons and 5–6 ml for 15 mm balloons). With an insertion length of 30 cm the majority of balloons will be placed in aortic zone III (below the renal arteries, but above the aortic bifurcation) [10]. In our routine, we also introduced balloon deflation every 10–15 minutes. The balloon was deflated for approximately one minute before it was re-inflated.

During uterine artery embolization, both arteries were selectively catheterized with a 4 or 5 French catheter, and in most cases medium sized (approximately 2 x 2 mm) gelatin sponge particles were used. In a majority of cases the femoral artery was sealed after removal of the introducer by using the 8 or 6 French Angio-Seal device (St Jude Medical, Minnetonka, MN, USA).

### Statistical analyses

Data from all eligible patients were analyzed. Continuous variables are shown as mean ± SD, and ordinal variables as median (range). Selected bivariate relationships were examined with the Spearman rank correlation test. A *P* value of < 0.05 was considered statistically significant. Analyses were conducted with IBM SPSS Statistics version 23.0.

## Results

### Demographics and clinical characteristics

Thirty-six patients (age 33.4 ± 5.9) were included in this study. Available demographics and clinical characteristics were obtained from the medical records (Table 2). In all patients, the use of REBOA resulted in an immediate and significant rise in systolic blood pressure (32 ± 22 mmHg), achieving hemodynamic stability. All insertions of the catheter for the occlusion balloon were performed in the OR, however, we have no records of the time from the emergency call to the balloon catheter insertion started.

**Table 2:** Patient demographics and clinical characteristics available from medical records.

Age, years	33 ± 6
Balloon insertion at the OR, %	100
Failed REBOA, %	0
Pre SBP, mmHg	75 ± 21
Post SBP, mmHg	106 ± 15
SBP rise, mmHg	32 ± 22
Balloon deflations, no	1 (0–5)
Balloon occlusion time, min.	33 ± 6
Total bleeding, L	5.2 ± 3.0
Blood transfusions, IU	9 (1–39)
Plasma, IU	6 (2–27)
Thrombocyte transfusions, IU	2 (0–36)
Curettage, no (%)	17 (47)
Intrauterine balloon, no (%)	7 (19)
Hysterectomy, no (%)	16 (44)
Embolization, no (%)	17 (47)

Data are presented as means ± SD, median (range) or no (%).

Six REBOA procedures resulted in complications. Five of them were related to local thrombus formation in the iliac or femoral artery, and all five occurred when using 11 cm introducers. Two of these patients had a surgical thrombectomy and three patients were treated with low-molecular heparin. All five patients recovered successfully. One patient, as previously reported [9], had an aortic tear that was treated surgically and recovered without any permanent sequelae. There were no complications regarding the insertion of the J-curved guide wire or the balloon catheter. The shorter introducer length (11 cm) was strongly associated with arterial thrombus formation (*ρ* = 0.50, *P* = 0.002). In addition, there was a strong negative correlation between uterine artery embolization and hysterectomy (*ρ* = -0.50, *P* = 0.002).

## Discussion

PPH is one of the leading causes of maternal mortality and morbidity.[2] Studies have suggested that many deaths associated with PPH could be prevented with prompt recognition and more rapid and adequate treatment [11,12]. Several studies have also noted an increase in PPH in high-resource countries, including Australia and Norway [13,14]. The use of expensive blood products may also be reduced by the use of REBOA, this is especially important in low-resource countries. The current study is the first series with systematic analysis on the subject of fluoroscopy-free REBOA for controlling life threatening PPH. REBOA elevated SBP in the setting of hemorrhagic shock in severe PPH and gained time for further evaluation and treatment, such as uterine artery embolization [15]. REBOA appears to be a quite safe procedure, and in our series no patients died from REBOA-related complications.

The main causes of PPH include uterine atony after childbirth, placenta adhesion, soft birth canal laceration, and coagulation abnormalities, the first by far being the most common reason [16]. Hysterectomy is an effective method to stop PPH, however, in combination with REBOA there is more time to wait for the effects of drugs given to induce contraction of the atonic uterus. If bleeding continues, uterine artery embolization should be considered. In combination with REBOA, this interventional technique can rapidly stop bleeding and preserve the reproductive function of the patient. This fact explains the correlation between uterine artery embolization and hysterectomy in our series.

There are several reports of using fluoroscopy for balloon positioning, however, fluoroscopy is time consuming and rarely available in the obstetric OR and settings with limited medical infrastructure. Guilani *et al*. have even shown that ultrasonography alone is safe and accurate for positioning and deployment of the balloon catheter [17]. Also, appropriately trained medical personnel with sufficient knowledge of the risk factors and procedures associated with an endovascular approach (e.g. interventional radiologists, anesthesiologists, and vascular surgeons) are often available during emergency situations. A recent review showed that from 16 fluoroscopy-free REBOA studies there was only one serious placement-related complication (aortic tear) [9], and precautions have now been taken to avoid this issue through reduction of the balloon size. Therefore, based on the low complication rate in previous and the present study, we recommend balloon positioning without the use of fluoroscopy.

In trauma patients there are several studies that have reported no vessel injuries caused by the femoral artery introducer or balloon catheter [18,19], however, a study by Saito et al. included two major complications with lower limb ischemia [20], 10 French introducers were used in these cases, in contrast to the present study where 6–8 French introducers were used. Complications in the present study were related to local iliac artery thrombus formation, which was significantly correlated to shorter introducer lengths (11 vs. 23 cm). Due to the sinuous iliac arteries it is possible that short introducers may affect the iliac vessel wall and thereby facilitate thrombus formation, while longer introducer lengths, more often will reach the abdominal aorta.

In the present study, no complications related to balloon occlusion time were observed, however, occlusion time was limited (33.3 ± 5.9 min.). In experiments with swine, Morrison *et al*. reported that a longer inflation time induces an inflammatory response through the release of interleukin 6, increases the incidence of adult respiratory distress syndrome, and increases the use of vasopressors [21]. A recent study showed that a REBOA duration of 60 min. in a swine model was well tolerated [22]. Furthermore, it is possible that the balloon sometimes ends up in the aortic zone II with the potential of occluding visceral and/or renal arteries. In a study of laparoscopic partial nephrectomy, a warm ischemia time of 40 min. was well tolerated before a significant decrease in renal function occurred [23].

Short balloon deflations allow reperfusion of the extremities and thereby reduces distal ischemia and the risk of thrombosis in non-heparinized patients. In the present study, repetitive deflations every 10–15 min. (each < 1 minute duration) were performed with an immediate deflation and balloon retraction after hemorrhage control. It is reasonable to believe that a combination of a short total occlusion time and repetitive balloon deflations probably will facilitate a minimum of ischemic complications. In addition, the balloon deflations gave time to evaluate if the patient was still bleeding. In a porcine hemorrhagic shock model, Russo *et al*. showed that partial balloon occlusion of the aorta (pREBOA) resulted in more physiologically tolerable hemodynamic and ischemic changes compared with complete occlusion [24]. Therefore, we believe it is important with a standardized procedure in REBOA to include either a balloon deflation regime or pREBOA.

The development of new devices, which do not require an oversized introducer, is likely to reduce not only complications but also time to occlusion [25]. In the last couple of years 7 French balloon catheters and introducers have been clinically available. Recently in Norway, a 15 mm diameter PTS-X balloon has been used in a few REBOA cases, which only require a 6 French introducer. Our experience is that in a hypovolemic PPH patient, a 15 mm balloon is sufficient to occlude or partially occlude the infra-renal aorta [26], and in this way stabilize the hemodynamic emergency situation.

### Limitations

Limitations in this study include particularly the small number of evaluated patients (n = 36), and that it is based on a retrospectively applied definition. However, to our knowledge, this is the largest study so far. Furthermore, a randomized controlled trial and/or the use of propensity scores is difficult to perform in the acute care setting. Additional multicenter studies are required to determine the effectiveness of REBOA.

## Conclusions

The present study indicates that fluoroscopy-free REBOA in an emergency situation can be performed with a high degree of technical success, to achieve hemodynamic stability, and to reduce mortality and morbidity associated with severe PPH.

During severe PPH, the combination of REBOA with uterine artery embolization should be considered to prevent unnecessary hysterectomies and to preserve the reproductive function of the patient.

Since adverse effects of invasive procedures cannot be avoided, the acquisition of standard procedures and safety implementation of REBOA is important.
